# A dual-transformable MgGa-MOF nanoplatform for HCC therapy via lactate metabolism blockade and immune reactivation

**DOI:** 10.1186/s12951-026-04356-8

**Published:** 2026-04-05

**Authors:** Yajie Li, Yingying Wei, Shaoshi Ma, Feng Li, Xianwei Meng, Shiping Yu

**Affiliations:** 1 Shanxi Province Cancer Hospital/ Shanxi Hospital Affiliated to Cancer Hospital, Chinese Academy of Medical Sciences/Cancer Hospital Affiliated to Shanxi Medical University, Taiyuan, China; 2 Medical Imaging Department, Shanxi Academy of Medical Sciences, Shanxi Medical University, Taiyuan, 030001 China; 3 State Key Laboratory of Cryogenic Science and Technology, Technical Institute of Physics and Chemistry, Laboratory of Controllable Preparation and Application of Nanomaterials, Technical Institute of Physics and Chemistry, Chinese Academy of Sciences, Beijing, 100190 China

**Keywords:** Microwave therapy, Microwave-responsive materials, Immune metabolism, Lactate metabolism modulation, Immune reactivation, Magnesium ions (Mg²⁺), Hepatocellular carcinoma

## Abstract

Microwave ablation (MWA) has emerged as one of the preferred modalities for treating hepatocellular carcinoma (HCC). However, its therapeutic efficiency is restricted by lactate accumulation after MWA. Lactate serves as a metabolic fuel for residual tumor cells, as well as acidifies the tumor microenvironment (TME) and impairs immune function, thereby fostering tumor recurrence and metastatic dissemination. Herein, we designed a dual-transformation strategy that turns metabolic fuel into metabolic burden and immunosuppressive pressure into power, implemented via bimetallic MOF-based nanoplatform (Dis@MgGa-MOF@TD/FA, DMGTF NCs), to counteract microwave-induced lactate elevation, reactivate immune activity and suppress primary tumor growth and metastatic progression. Specifically, after intravenous administration, folic acid (FA)-modified DMGTF accumulates in HCC, where microwave irradiation opens the 1-Tetradecanol (TD) gate to release diclofenac sodium (Dis). The released Dis suppresses MCT4-mediated lactate efflux, thereby disrupting lactate-driven energy supply and reshaping the TME. Meanwhile, microwave-activated DMGTF generates abundant ROS to impair mitochondrial lactate oxidation, thereby promoting intracellular lactate accumulation and inducing metabolic stress. Moreover, framework-derived Mg²⁺ restarts T cells, boosts proliferation, and augments IFN-γ secretion, converting immunosuppressive “pressure” into antitumor “power”. As a result, DMGTF NCs combined with MW achieve excellent therapeutic effects in a model of hepatocellular carcinoma and lung metastasis. This MOF-based dual-transformation strategy provides a promising solution to the long-standing challenge of post-MWA tumor relapse and dissemination, offering new insights into the effective control of liver cancer.

**Graphical abstract:**

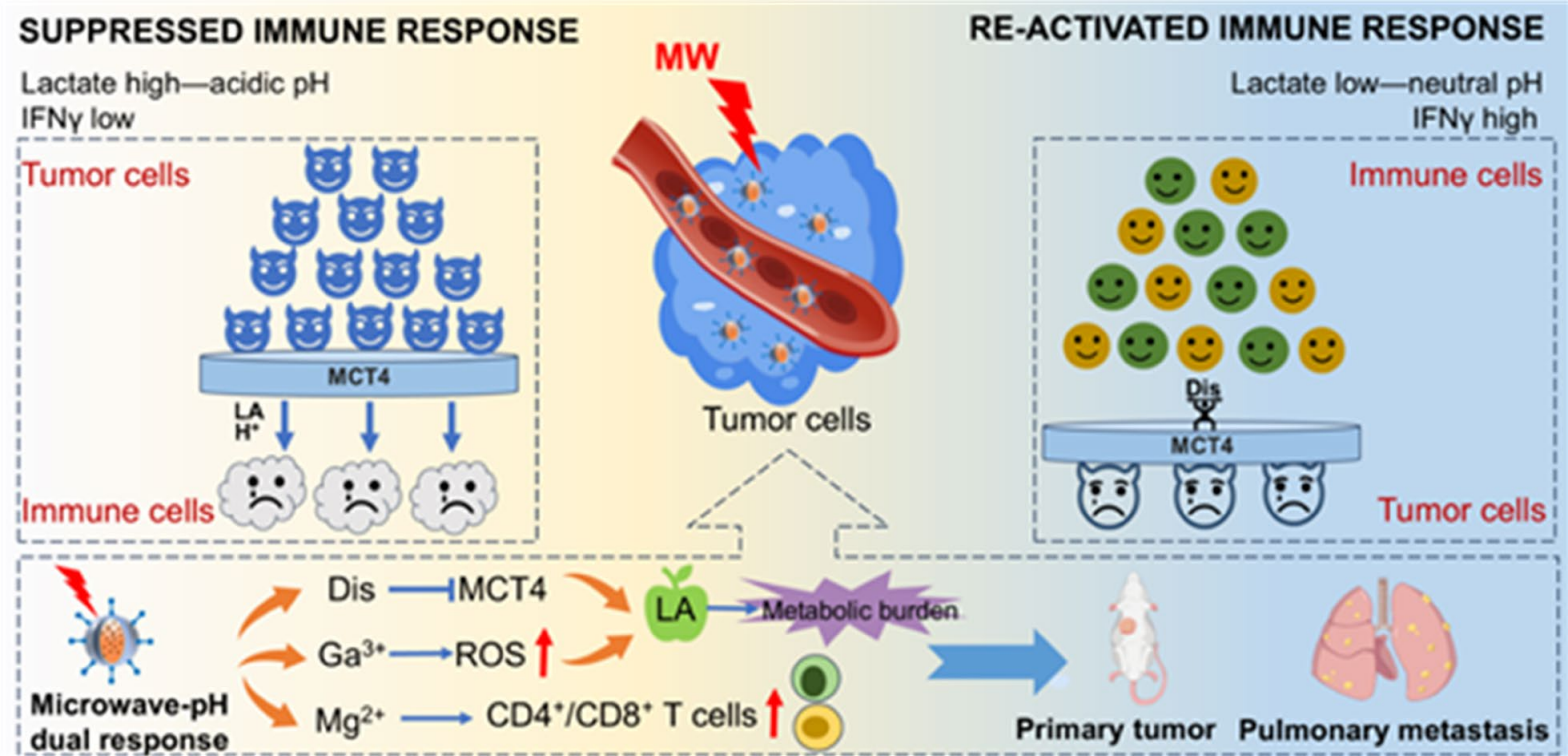

**Supplementary Information:**

The online version contains supplementary material available at 10.1186/s12951-026-04356-8.

## Introduction

Hepatocellular carcinoma (HCC) remains a major global health burden with rising incidence and mortality. Although surgical resection and systemic therapies are available, effective local control remains challenging [1–3]. Microwave ablation (MWA) has emerged as a minimally invasive treatment modality for HCC, offering precise thermal destruction with reduced procedural morbidity [4–6]. Recent advances in nanotechnology have further expanded the therapeutic scope of MWA by enhancing intratumoral energy deposition and enabling modulation of the tumor microenvironment [7–10]. However, complete tumor eradication by MWA remains challenging due to the formation of sublethal zones generated by intrinsic thermal gradients, where residual tumor cells survive thermal stress and undergo profound metabolic reprogramming characterized by enhanced glycolytic flux and excessive lactate production [11–13].

Accumulated evidence implicates the diverse and substantial influence of lactate in driving tumor recurrence and metastasis [14–16]. Excess lactate not only serves as a fuel to sustain residual tumor cell proliferation but also contribute to the acidification of the tumor microenvironment (TME) [17–19]. This acidic microenvironment imposes substantial pressure on cytotoxic T cells, suppressing immune effector cell proliferation and inducing immune cell de-differentiation, to promote immune evasion and tumor spread [20, 21]. Therefore, increasing efforts have been directed toward intervening in lactate metabolism to disrupt tumor metabolic adaptation and simultaneously alleviate the immunosuppressive tumor microenvironment [22–25]. For example, recent work using PDK1-degrading PROTACs has been shown to modulate aerobic glycolysis and induce immunogenic cell death, and Qian and colleagues demonstrated that MCT4-mediated lactate export impairs antitumor immune responses [26, 27]. Notably, MCT4 inhibition has been shown in multiple cancer models to reduce lactate efflux and enhance immune and oxidative stress responses [28, 29].

However, inhibiting lactate efflux via MCT4 blockade fails to comprehensively disrupt tumor metabolism and reactivate immunodynamics [30–32]. First, even when lactate efflux is blocked, tumor cells can reroute intracellular lactate into mitochondrial metabolism, continuing to generate fuel and undermining the effectiveness of lactate-targeted interventions [33]. Furthermore, although extracellular lactate levels have been partially reduced, immune cells chronically exposed to a suppressive TME rarely regain full cytotoxic function, leading to incomplete immune reactivation and failure of antitumor immune surveillance [34, 35]. Therefore, it is crucial to develop new strategies to comprehensively block lactate metabolism and restart dysfunctional immune cells in parallel with MWA.

Given the dual challenge, we propose a dual-transformation strategy that converts metabolic fuel into burden and immunosuppressive pressure into immune power. Specifically, metal–organic frameworks (MOFs) were employed as microwave-sensitizing nanoplatforms [36–38]. On one hand, they facilitate the delivery of an MCT4 lactate transporter inhibitor to obstruct lactate efflux and disrupt intercellular energy transfer in tumor cells [39, 40]. On the other hand, the microwave-responsive ROS generated by MOFs induces mitochondrial damage in tumor cells, inhibiting lactate oxidation and utilization [41–43]. This dual blockade effectively lowers extracellular lactate while increasing intracellular lactate, converting lactate from a metabolic fuel into metabolic burden Importantly, the metal ions within MOFs potentiate T cell proliferation and enhance effector functions, thereby transforming immunosuppressive pressure into immunostimulatory activity and eliciting systemic antitumor immunity [44–46]. As such, the introduction of MOF-based nanoplatforms provides a solid foundation for implementing the dual-transformation strategy, which may further address recurrence and metastasis in liver cancer.

**Scheme 1:**
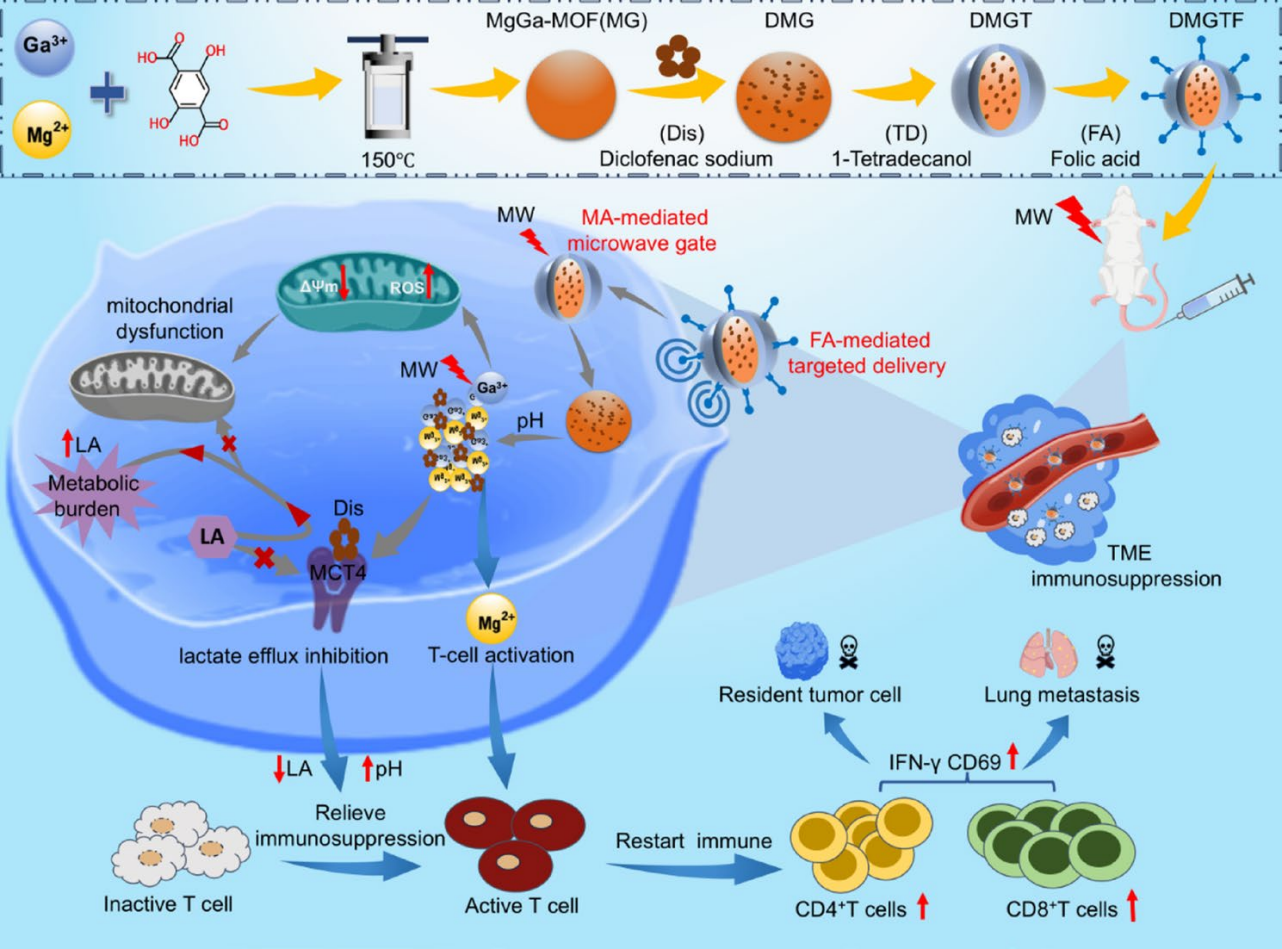
Schematic diagram of DMGTF NCs synthesis and anti-tumor therapy

In this work, a liver-targeted, microwave-and pH-dual-responsive, degradable bimetallic metal–organic framework (Dis@MgGa-MOF@TD/FA, DMGTF NCs) has been constructed to co-deliver diclofenac sodium (Dis), the previously described MCT4 inhibitor. DMGTF NCs exhibits the following important advantages (Scheme 1): (1) Folic acid (FA) endows the nanoplatforms with liver cancer targeting and enhances cellular endocytosis ensuring preferential accumulation of DMGTF within tumor cells [47–49]. (2) 1-Tetradecanol (TD), forming the shell of DMGTF and serving as microwave thermal-responsive gate, enables controllable release of Dis under microwave irradiation, achieving precise delivery to tumor cells interiors [50]. (3) Dual-pathway lactate blockade is achieved, in which Dis inhibits MCT4-mediated lactate export and synergizes with microwave-induced ROS generated by Ga^3+^ to damage mitochondrial function and block lactate oxidation, ultimately converting lactate from a tumor fuel into unusable metabolic burden and collapsing the survival foundation of residual tumor cells [51, 52]. (4) Mg²⁺ reverses T-cell exhaustion, promotes proliferation, and enhances the secretion of effector molecules IFN-γ, thereby reactivating immune surveillance and inducing tumor cell killing [53–55]. DMGTF once again implements a transformation strategy that converts the immunosuppressive pressure of the TME into a driving force for potent antitumor immunity. Under microwave irradiation, DMGTF exhibits significant therapeutic effects in both liver cancer and lung metastasis models. Therefore, the successful construction of the dual-transformation nanoplatforms Dis@MgGa-MOF@TD/FA (DMGTF NCs) provides a paradigm for clinical to address tumor recurrence and metastasis after microwave treatment through effectively inhibiting tumor lactate metabolism, reactivating immune cell function, and inducing durable immune memory in vivo.

## Results and discussion

### Rationale for constructing DMGTF nanoplatform

We utilized MOF-based nanoplatforms to overcome the low bioavailability and poor tumor-targeting of lactate transporter inhibitors. To enhance tumor targeting and cellular uptake, folic acid (FA) was anchored onto the surface, while 1-tetradecanol (TD) was incorporated as a microwave-responsive switch to enable controlled release of diclofenac sodium (Dis). By carefully selecting the metal nodes and organic linkers, the MOFs were further tailored to integrate metabolic interference and immune regulation. Specifically, Ga³⁺ was incorporated to enhance microwave-triggered ROS generation, thereby inducing mitochondrial damage and further inhibiting lactate metabolism. Mg²⁺ provided excellent biocompatibility and modulated the immune microenvironment, promoting T cell activation and proliferation. Taken together, the Mg-Ga-MOF nanoplatforms functionalized with TD and FA constitute a multifunctional platform that enables the implementation of our dual-transformation strategy.

### Synthesis and characterization of DMGTF

MgGa-MOF (MG) was synthesized via a hydrothermal route using Ga (NO)_3_· xH2O, Mg (NO₃) ₂·6 H₂O, and terephthalic acid as precursors. Diclofenac sodium (Dis) was subsequently incorporated into MG through orbital shaking, yielding the formulation designated as DMG. Thereafter, 1-Tetradecanol (TD), serving as a microwave-responsive molecular switch, together with folic acid (FA), providing tumor-targeting capability, was anchored onto the DMG surface via a vacuum-assisted adsorption process to obtain the multifunctional nanoplatform, DMGTF NCs. SEM and TEM images revealed that the MG nanoparticles exhibited a uniform spherical morphology with a relatively homogeneous size distribution (Fig. 2A, B). Dynamic light scattering (DLS) analysis showed a moderate increase in hydrodynamic diameter after surface modification, with the average size increasing from 176.6 nm to 269.3 nm (Fig. 2C). Meanwhile, ζ-potential measurements displayed a pronounced shift in surface charge, with the values of 11.73mV for MG, -2.11mV for DMG, -5.82mV for DMGT, -12.47mV for DMGTF, collectively confirming the successful surface functionalization (Fig. 2D). Energy-dispersive X-ray spectroscopy (EDS) confirmed the presence of Ga, Mg, C, and O elements with weight percentages of 15.78%, 14.22%, 49.14%, and 20.86%, respectively, verifying the successful incorporation of Mg and Ga into the MOF framework (Fig. 2E). Fourier transform infrared (FTIR) spectra further validated the stepwise functionalization of DMGTF (Fig. 2F). For pristine MgGa-MOF, characteristic bands at approximately 1535 cm⁻¹ and 1397 cm⁻¹ corresponded to the asymmetric and symmetric stretching vibrations of the coordinated carboxylate groups from terephthalic acid. After folic acid modification, an additional C = O stretching vibration (1600–1650 cm⁻¹) became discernible, leading to an overall enhancement and broadening of the absorption band within 1569–1689 cm⁻¹, confirming the successful conjugation of folic acid onto the MOF surface. Moreover, the emergence of the C-Cl stretching vibration of diclofenac sodium at 752 cm⁻¹ and the -CH₂- stretching band of 1-Tetradecanol (TD) at 2923 cm⁻¹ further verified the coexistence of both components in the final composite. X-Ray Diffraction (XRD) patterns indicated that MgGa-MOF retained a well-defined crystalline framework after modification (Fig. 2G). Collectively, these results demonstrate the successful construction of the multifunctional nanoplatform DMGTF.

**Fig. 1:**
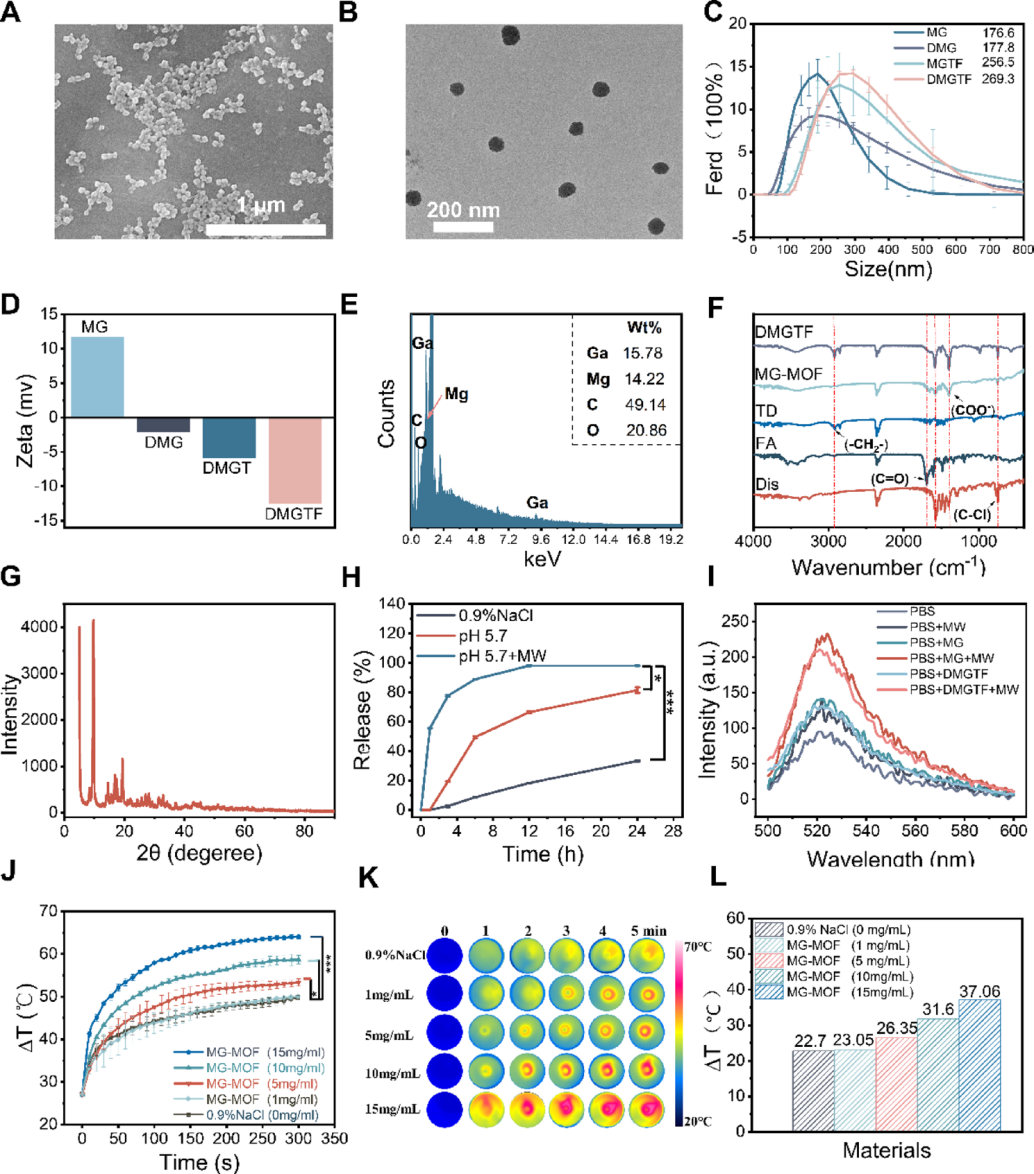
Characterization and Performance of DMGTF nanoplatforms. (**A**) SEM image of MG nanoparticles. (**B**) TEM image of MG nanoparticles. (**C**) Hydrodynamic diameter distributions of MG, DMG, MGTF and DMGTF nanoparticles. (**D**) ζ-Potential variations before and after surface modification. (**E**) Energy-dispersive X-ray spectroscopy (EDS) analysis of MGTF. (**F**) FTIR spectra of FA, TD, Dis, MG-MOF and DMGTF. (**G**) XRD pattern of MG. (**H**) Drug release profiles of DMGTF under various conditions. (**I**) Reactive oxygen species (ROS) generation of MG and DMGTF. (**J**) Microwave-induced temperature elevation curves of MG at different concentrations. (**K**) Temperature variations of MG under microwave irradiation. (**L**) Temperature increments of MG under different conditions. *p < 0.05, **p < 0.01, ***p < 0.001 and ****p < 0.0001

### Biodegradability evaluation and drug release of MGTF

To assess environmental responsiveness, MG was dispersed in PBS at pH 7.4 and pH 5.7 to simulate physiological and TME, respectively. TEM imaging (Fig. S1) revealed that the nanoparticles maintained stable morphology at pH 7.4 for up to 9 h. In contrast, under acidic conditions (pH 5.7), the number of intact nanoparticles gradually decreased from 3 h to 9 h, with evident structural degradation observed at 9 h, indicating acid-triggered biodegradability. Notably, microwave irradiation at pH 5.7 induced a rapid collapse of the spherical structure within 10 min and almost complete degradation after 30 min, demonstrating that microwave exposure further accelerates the breakdown of the 1-tetradecanol and MgGa-MOF, thereby enabling an effective microwave-triggered on–off release behavior.

**Fig. 2:**
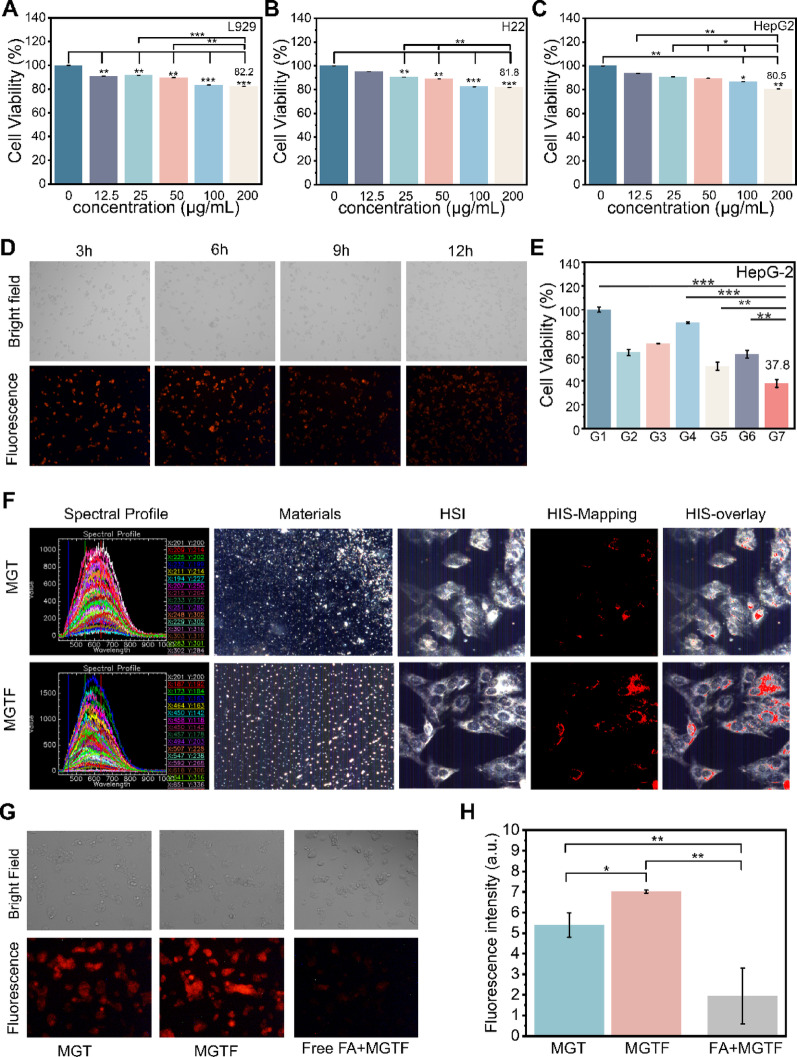
In vitro evaluation of DMGTF nanoplatforms. (**A-C**) Dose-dependent cytotoxicity of MGTF toward L929, H22, and HepG2 cells after 24 h incubation. (**D**) Confocal fluorescence microscopy images showing the time-dependent internalization of MGTF by cells. (**E**) Therapeutic efficacy of DMGTF nanoplatforms against tumor cells under different treatment conditions. (**F**) Hyperspectral imaging of MGT and MGTF after cellular incubation. (**G**) Fluorescence intensity of MGT, MGTF, and MGTF after folic acid (FA) blocking treatment. (**H**) Quantitative analysis of fluorescence intensity. * *p* < 0.05, **p < 0.01, ***p < 0.001 and ****p < 0.0001

Diclofenac sodium (Dis), a lactate transporter inhibitor, was successfully encapsulated within DMGTF. UV-vis spectroscopy exhibited a characteristic absorption peak at 276 nm, which was used for quantitative calibration (Fig. S2a, b). The encapsulation efficiency and drug loading capacity were calculated to be 34.4% and 20.5%, respectively. Drug release profiles (Fig. 2H) demonstrated cumulative Dis release rates of 33.3% in neutral PBS, 81.5% in pH 5.7 PBS, and 98.1% in pH 5.7 + MW PBS conditions. Notably, after 6 h, the cumulative drug release under pH 5.7 + MW conditions were 10.3-fold and 5.75-fold higher, respectively, than under the neutral environment and pH 5.7 conditions, indicating clear microwave-and pH-dual-responsive release behavior.

### Further evaluation of the microwave responsiveness of MgGa-MOF

A central objective of this work was to construct a metal-organic framework with both microwave heating and dynamic therapeutic functionalities as a foundation for noninvasive microwave therapy. The microwave dynamic effect of MgGa-MOF was investigated by monitoring reactive oxygen species (ROS) generation using DCFH-DA (Fig. 2I). Minimal ROS signals were observed in the PBS and PBS + MW groups, while PBS + MG exhibited only a slight increase. In sharp contrast, the PBS + MG + MW group showed markedly enhanced ROS production, with fluorescence intensities 2.46-, 1.67-, and 1.64-fold higher than those of PBS, PBS + MW, and PBS + MG, respectively. After modification of MgGa-MOF with 1-Tetradecanol and folic acid, the microwave-induced ROS generation capability slightly decreased compared with that of pristine MgGa-MOF, while the material still retained considerable ROS-producing activity under microwave irradiation. Additionally, as shown in Fig. 2J-L, the temperature rise displayed a clear positive correlation with particle concentration, reaching 23.05, 26.35, 31.60, and 37.06 °C at 1, 5, 10, and 15 mg mL⁻¹, respectively, compared with 22.7 °C for the control. After modification with 1-tetradecanol and folic acid, DMGTF exhibited a slightly reduced microwave heating performance compared with MG, while still maintaining a similar concentration-dependent temperature elevation behavior (Figure S3a, b).The corresponding temperature differentials (0.35, 3.65, 8.90, and 14.36 °C) confirmed a distinct concentration-dependent microwave heating effect, underscoring the excellent microwave-responsive capability of MgGa-MOF and its potential as a microwave hyperthermia agent. These findings demonstrate that MgGa-MOF can act both as a microwave sensitizer for microwave-induced heating and ROS generation, thereby enabling synergistic microwave thermal and dynamic therapeutic effects.

### Biocompatibility of MgGa-MOF

**Fig. 3:**
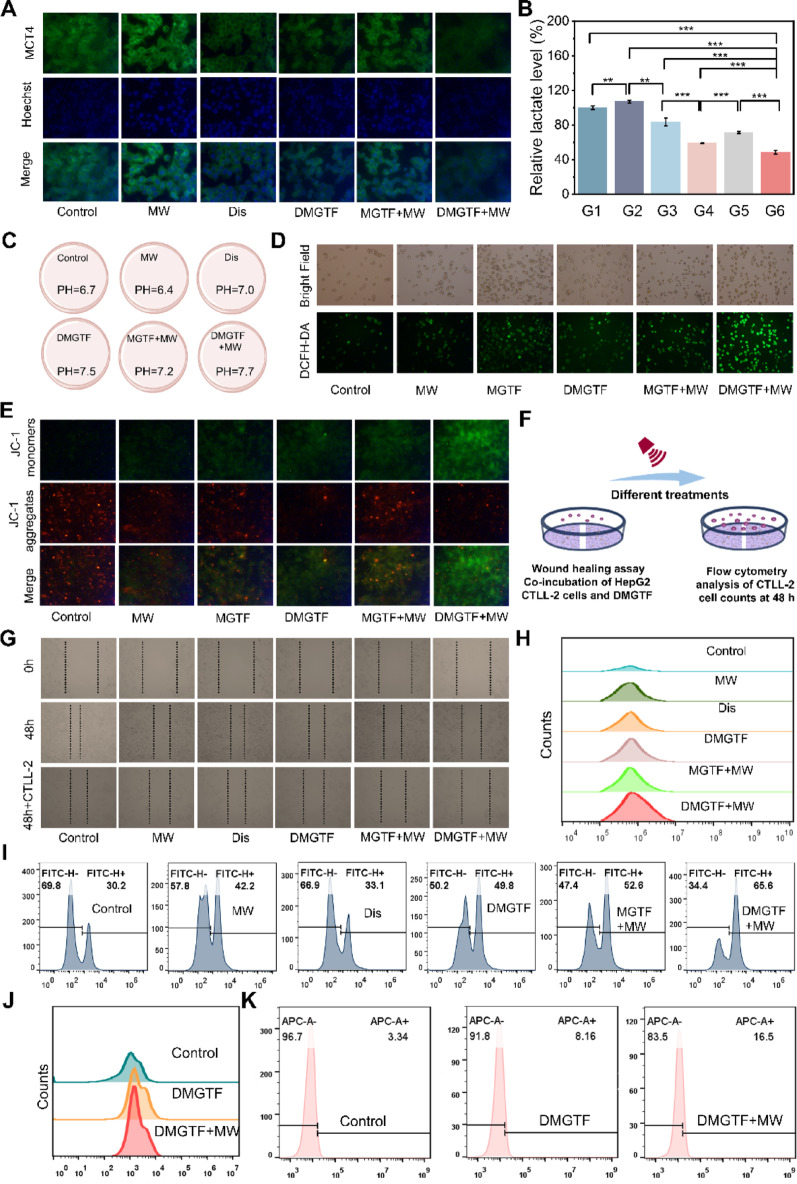
In vitro evaluation of DMGTF. (**A**) Immunofluorescence images of MCT4 protein (green) and Hoechst-stained nuclei (blue). (**B**) Extracellular lactate concentrations under different treatment conditions. (**C**) Extracellular pH under different treatments. (**D**) Fluorescence images showing intracellular ROS generation in HepG2 cells under various conditions. (**E**) Changes in mitochondrial membrane potential of HepG2 cells after different treatments. (**F**) Schematic illustration of DMGTF acting on HepG2 tumor cells and CTLL-2 immune cells. (**G**) Wound healing assay of HepG2 cells under different conditions. (**H**) Flow cytometry analysis of immune cell proliferation under tumor cell–material–immune cell co-culture conditions. (**I**) Flow cytometry analysis of IFN-γ expression in immune cells under tumor cell–material–immune cell co-culture conditions. (**J**) Flow cytometry analysis of CTLL-2 proliferation after treatment with DMGTF alone. (**K**) Flow cytometry analysis of IFN-γ expression in CTLL-2 cells after treatment with DMGTF alone. The mean ± standard deviation (SD) data are presented, with *n*  ≥3, *p < 0.05, **p < 0.01 and ***p < 0.001

In vitro studies demonstrated that MgGa-MOF acts as a versatile microwave sensitizer with strong thermal and dynamic effects, while also serving as a pH-responsive drug carrier, underscoring its potential for tumor therapy. To further evaluate its biosafety, comprehensive biocompatibility studies were performed. Cytotoxicity assays showed that after 24 h of incubation with L929, H22, and HepG2 cells, the cell viabilities remained at 82.2%, 81.8%, and 80.5%, even at concentrations up to 200 µg mL⁻¹ (Fig. 3A-C). Similarly, the complete nanoplatform DMGTF also exhibited low cytotoxicity toward these cells at a concentration of 100 µg mL⁻¹ (Figure S4). In vivo acute toxicity tests in mice revealed no significant changes in body weight (Figure S5a) or hematological parameters (Figure S5b). The major organs (heart, kidney, liver, lung, and spleen) were collected for sectioning and hematoxylin and eosin (H&E) staining. No apparent lesions were observed in either the experimental groups (50 mg kg ^− 1^,75 mg kg^ − 1^, 100 mg kg^ − 1^) or the control group (Figure S6). Collectively, these results confirm the excellent biocompatibility and biosafety of MgGa-MOF both in vitro and in vivo, supporting its potential as a multifunctional therapeutic nanocarrier.

### Cellular uptake and tumor-targeting efficiency of DMGTF

Encouraged by its excellent biosafety profile, the therapeutic performance of DMGTF was next evaluated in vitro. Considering that nanoparticle uptake depends on incubation time, endocytosis assays were conducted to identify the optimal internalization window. Rhodamine-loaded MgGa-MOF allowed fluorescence tracking, and confocal laser scanning microscopy revealed maximal intracellular accumulation at 6 h (Fig. 3D), which was therefore used for subsequent experiments. Given that folic acid is a widely used targeting ligand due to the overexpression of folate receptors in hepatocellular carcinoma (HCC) cells compared to normal liver tissue, FA was successfully conjugated onto the MgGa-MOF surface to achieve tumor targeting. Hyperspectral microscopy provided more detailed evidence confirming that FA significantly enhances cellular targeting. First, MgGa-MOF nanoparticles with or without FA modification were characterized to establish a spectral library for both nanomaterials (Fig. 3F–Materials). This library was then applied to map the hyperspectral images of HepG2 cells following nanoparticle internalization. In Fig. 3F, HSI shows the hyperspectral images of cells incubated with the two nanoparticle formulations, whereas the HSI-Mapping panel uses red coloration to indicate the relative abundance of nanoparticles within the cells. The HSI-Overlay panel visualizes the spatial overlap between nanoparticle signals and cellular structures. After 6 h of incubation, both FA-modified (MGTF) and unmodified (MGT) nanoparticles were internalized; however, the mapped spectral intensity of MGTF was markedly higher, suggesting more efficient intracellular enrichment. This enhancement is attributed to FA-mediated receptor recognition and active endocytosis rather than passive uptake alone. To further verify the role of folate receptor recognition, a competitive inhibition experiment was performed by pre-incubating HepG2 cells with excess free FA (1 mM) to block folate receptors. As shown in Fig. 3G, FA-modified nanoparticles exhibited significantly higher intracellular fluorescence than non-modified nanoparticles, whereas the fluorescence signal markedly decreased after FA blocking. Quantitative analysis of fluorescence intensity using ImageJ further confirmed this trend (Fig. 3H), indicating that the enhanced cellular uptake is mediated by folate receptor-dependent endocytosis. Collectively, these findings demonstrate that FA conjugation endows MGTF with superior targeting capability toward hepatocellular carcinoma cells and significantly enhances cellular uptake, thereby providing a solid foundation for subsequent therapeutic investigations.

### In vitro therapeutic performance of DMGTF

The therapeutic efficacy of DMGTF was evaluated in H22 and HepG2 cells under various treatment conditions (Fig. 3E, S7). Seven groups were included: Control (G1), MW alone (G2), Dis (G3), MGTF (G4), MGTF + MW (G5), DMGTF(G6), and DMGTF + MW (G7). Using the control group (G1) as a reference, cell viability in the MGTF group (G4) remained highest among all treatments, consistent with earlier cytotoxicity results at 100 µg mL⁻¹ (> 80%). MW treatment alone (G2) reduced viability to ~60% in both cell lines. In the Dis group (G3), H22 and HepG2 cells exhibited viabilities of 62.8% and 71.4%, respectively, likely reflecting differences in drug sensitivity. Notably, combining MGTF with MW (G5) further decreased cell viability to ~ 50%, highlighting the contribution of microwave stimulation. DMGTF treatment (G6) reduced viability to ~60%, whereas the addition of MW (G7) further decreased viability to ~ 35%, demonstrating a synergistic effect of the nanomaterial, drug, and microwave irradiation. Collectively, these results indicate that DMGTF exhibits potent cytotoxicity under microwave irradiation, effectively inducing apoptosis and supporting its potential for in vivo tumor therapy.

### DMGTF under microwave irradiation blocks lactate transport and neutralizes the acidic TME

**Fig. 4:**
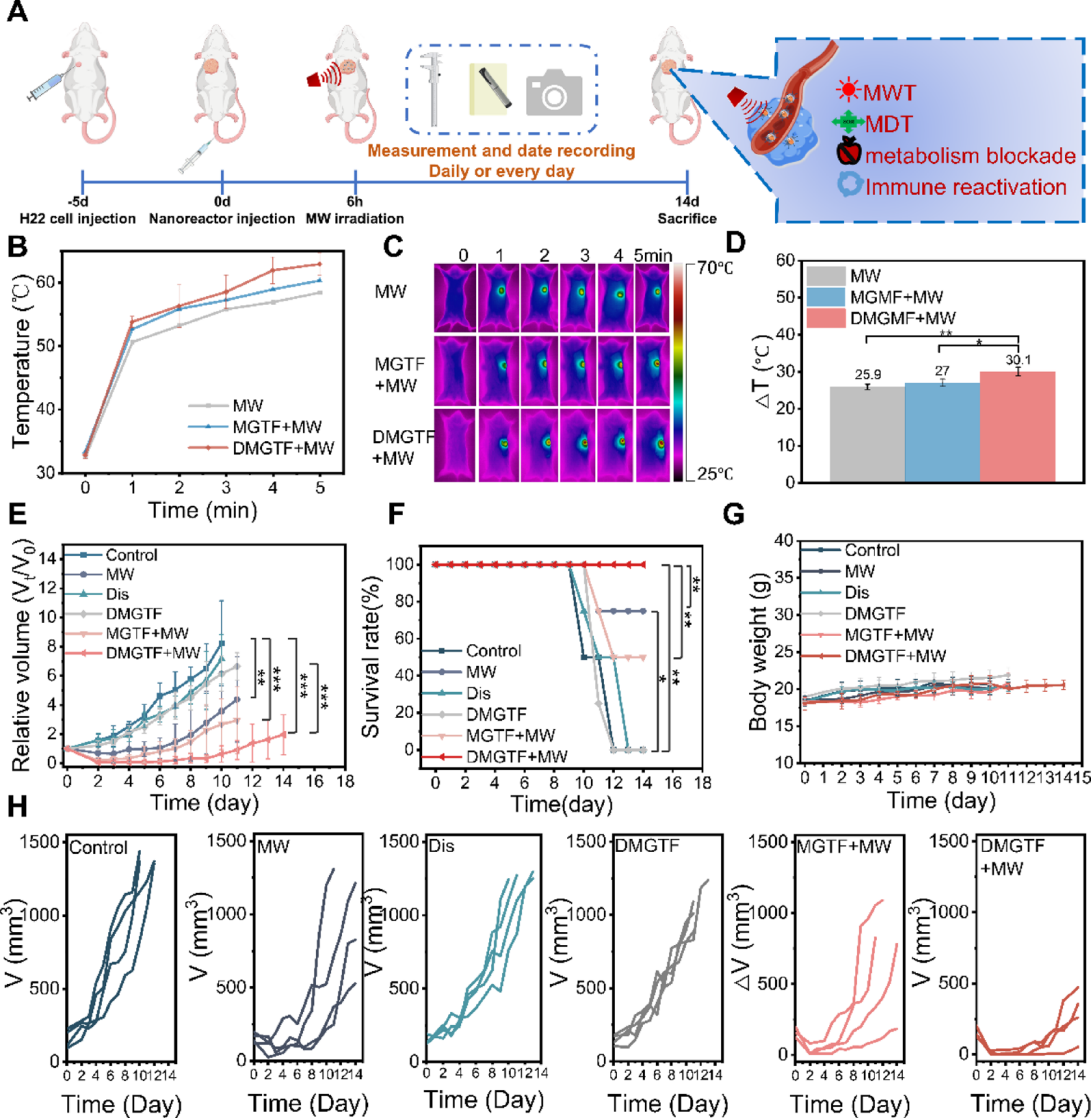
In vivo evaluation of DMGTF nanoplatforms. (**A**) Schematic illustration of the experimental design using H22 tumor-bearing mice, in which the left-side tumor was designated as the primary tumor for treatment. (**B**) Temperature elevation profiles during MW irradiation. (**C**) Representative infrared thermal images recorded during MW exposure. (**D**) Average temperature increments among different groups. (**E**) Relative tumor growth curves after various treatments. (**F**) Survival rates of mice under each treatment condition. (**G**) Body-weight variations during the treatment period. (**H**) Tumor-volume curves for each group (*n* = 4)

As shown in Fig. 4A, control cells exhibited basal MCT4 expression primarily localized on the plasma membrane and cytoplasm. Microwave (MW) exposure markedly enhanced MCT4 expression, consistent with MW-induced glycolytic activation. In contrast, both diclofenac sodium (Dis) and DMGTF treatments significantly suppressed MCT4 levels, with the most pronounced downregulation observed in the DMGTF + MW group. Consistent with these findings, lactate quantification (Fig. 4B) revealed that MW irradiation increased lactate secretion, whereas Dis and DMGTF notably reduced lactate efflux. The DMGTF + MW combination achieved the lowest extracellular lactate concentration, confirming the effective blockade of lactate transport under MW stimulation. Correspondingly, intracellular lactate analysis showed the opposite trend, with the DMGTF + MW group exhibiting the highest intracellular lactate level, further indicating lactate accumulation caused by inhibited lactate efflux (Figure S8). Restricting lactate efflux disrupts the intercellular metabolic exchange that normally functions as an energy shuttle, thereby converting lactate from a metabolic fuel into a metabolic burden. Medium pH measurements further substantiated these results. The control group showed a pH of 6.7, which further decreased upon MW treatment due to accelerated metabolism. In contrast, Dis treatment increased pH to 7.0 by suppressing MCT4-mediated lactate export. DMGTF and DMGTF + MW groups exhibited further alkalization, reaching a pH of 7.7 in the latter (Fig. 4C). This substantial neutralization of the acidic TME can be attributed to enhanced cellular uptake via FA-mediated targeting and efficient MCT4 inhibition induced by the drug-loaded MgGa-MOF under MW irradiation.

### DMGTF-induced mitochondrial dysfunction under microwave activation

Beyond blocking lactate efflux, converting lactate from a metabolic fuel into a burden product requires disrupting its mitochondrial utilization. DMGTF achieves this effect by inducing microwave-driven mitochondrial damage. Intracellular ROS generation was assessed using the DCFH-DA probe (Fig. 4D) [56]. MW exposure alone elevated ROS levels, while both MGTF + MW and DMGTF + MW treatments produced stronger fluorescence signals, with DMGTF + MW showing the most intense ROS accumulation. In contrast, MGTF and DMGTF without MW irradiation exhibited weak fluorescence, underscoring the indispensable role of MW activation in amplifying oxidative stress. Mitochondrial membrane potential (ΔΨm) was subsequently evaluated by JC-1 staining (Fig. 4E). Control, MW-only, and MGTF groups predominantly exhibited red fluorescence with minimal green signal, indicating intact mitochondrial function. Conversely, both MGTF + MW and DMGTF + MW groups showed markedly increased green fluorescence, signifying mitochondrial depolarization. Notably, partial retention of red fluorescence in the MGTF + MW group suggested transient hyperpolarization in some cells during MW stress. The DMGTF + MW group displayed the most pronounced decline in the red-to-green ratio, confirming severe mitochondrial damage and the strongest disruption of mitochondrial integrity. Collectively, these findings indicate that DMGTF, under MW irradiation, not only blocks extracellular lactate export but also suppresses mitochondrial energy metabolism through ROS-mediated depolarization, thereby reinforcing metabolic collapse in tumor cells. In this context, the metabolic pathway required for lactate oxidation is critically disrupted, further preventing its utilization and ultimately converting lactate from an energy substrate into an unusable metabolic burden.

### DMGTF enhances antitumor immunity in a tumor–immune cell coculture system

A coculture model of HepG2 tumor cells and CTLL-2 immune cells was established to evaluate the dual effects of DMGTF on tumor inhibition and immune activation (Fig. 4F). As shown in Fig. 4G, in the absence of immune cells, tumor cells in the Control and Dis groups exhibited rapid proliferation, while MW, MGTF + MW, and DMGTF + MW treatments progressively inhibited cell growth, with DMGTF + MW showing the strongest suppression. Upon the introduction of CTLL-2 cells, tumor inhibition was further enhanced across all groups, which can be attributed to the combined cytotoxic effect of DMGTF on tumor cells and its immunostimulatory influence on T cells. Flow cytometric analysis of suspended CTLL-2 cells (Fig. 4H) revealed a marked increase in immune cell proliferation, with the DMGTF + MW group reaching 375,912 cells per 300 µL medium approximately a 7.6-fold increase compared with the control. The inhibition of tumor cells can be attributed partly to the direct cytotoxic effects of DMGTF, MW irradiation, and activated immune cells. More importantly, DMGTF not only induces tumor cell death but also promotes the proliferation of immune cells, thereby establishing a positive feedback loop that further amplifies the antitumor response. Furthermore, immunophenotyping demonstrated that T cell activation markers IFN-γ and CD69 (Fig. 4I and Fig. S9) were most strongly upregulated in the DMGTF + MW group, surpassing those in MW, Dis and MGTF group. Taken together, DMGTF under microwave irradiation exerts synergistic antitumor effects by suppressing lactate metabolism, inducing ROS-mediated mitochondrial dysfunction, and enhancing immune cell activation, thereby relieving tumor immunosuppression and amplifying therapeutic efficacy. These combined actions hold strong potential for preventing tumor recurrence and metastasis following microwave therapy.

Although enhanced T-cell activity in the tumor–immune coculture system may partially result from microenvironmental relief, such as decreased lactate accumulation and elevated extracellular pH, it remains unclear whether DMGTF can directly stimulate immune cells. To clarify this issue, CTLL-2 cells were cultured with DMGTF in the absence of tumor cells. Flow cytometry analysis showed that the DMGTF + MW group exhibited significantly enhanced CTLL-2 proliferation compared with the control group (107,902 vs. 56,246 cells per 200 µL). Meanwhile, the expression levels of IFN-γ and CD69 were markedly elevated, with 4.94-fold and 6.98-fold increases, respectively (Fig. 4J, K, and S10). These results demonstrate that DMGTF under microwave irradiation can directly promote immune cell proliferation and activation, further supporting its role in converting immunosuppressive pressure into immune activation within the tumor microenvironment.

### In vivo tumor suppression of DMGTF

**Fig. 5:**
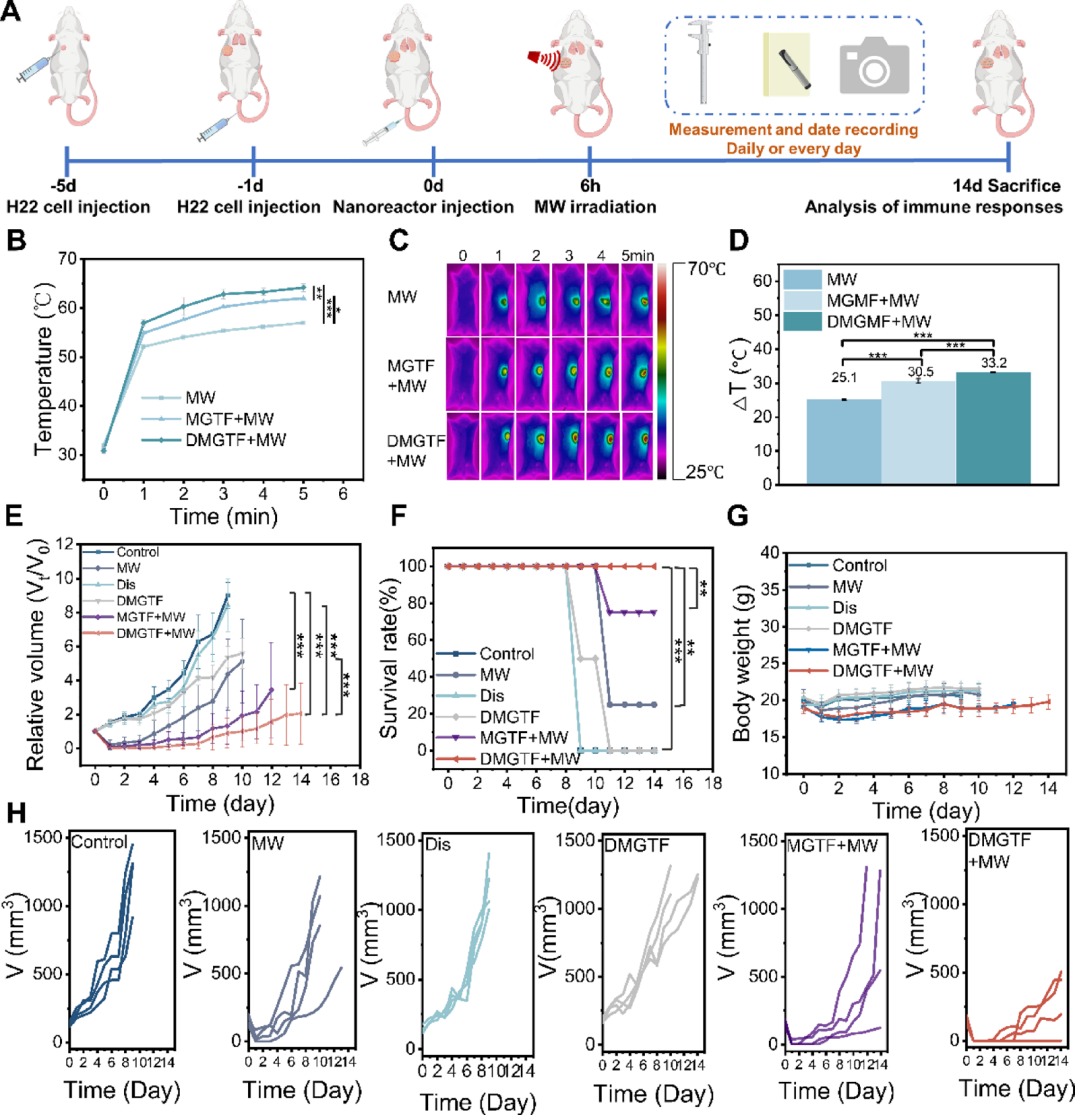
In vivo evaluation of DMGTF for lung metastatic tumor therapy. (**A**) Schematic illustration of the treatment strategy for metastatic tumors with DMGTF. (**B**) Temperature elevation curves of primary tumors during MW therapy. (**C**) Representative thermal images of primary tumors under MW irradiation. (**D**) Bar chart showing temperature differences among groups. (**E**) Relative growth curves of primary tumors after treatment. (**F**) Survival rates of mice under different treatments. (**G**) Body weight changes of mice after various treatments. (**H**) Tumor volume curves of each group (*n* = 4)

The in vivo antitumor efficacy of DMGTF was evaluated using an H22 subcutaneous syngeneic hepatocellular carcinoma model (Fig. 5A). Mice were intravenously injected with different formulations at 50 mg/kg, followed by microwave irradiation (1.8 W, 5 min) 6 h post-injection. As shown in Figs. 5B–D, tumor temperatures increased by 25.9 °C, 27.0 °C, and 30.1 °C in the MW, MGTF + MW, and DMGTF + MW groups, respectively. Statistical analysis of temperature elevation demonstrated that the DMGTF + MW group exhibited a significantly greater temperature rise than the MW group (*p* < 0.01), while both the MGTF + MW and DMGTF + MW groups showed markedly enhanced heating performance, confirming the pronounced microwave-sensitization effect of MgGa-MOF. Tumor growth curves (Fig. 5E) revealed that the tumor volume in the DMGTF + MW group was markedly smaller than that in all other groups throughout the 14-day observation period. Owing to the aggressive proliferation of H22 tumors, several mice in the Control and Dis groups reached humane endpoints and were euthanized on day 10, while the DMGTF, MW-only and MGTF + MW groups were terminated on day 11 according to ethical requirements. Notably, only the DMGTF + MW group completed the full 14-day therapeutic schedule. Consistently, survival analysis (Fig. 5F) demonstrated that DMGTF + MW treatment significantly prolonged survival compared with all other groups. These outcomes can be attributed to both the intrinsically high proliferative capacity of primary H22 tumors-resulting in slightly larger tumor sizes at baseline and the limited antitumor efficacy of Dis or MW alone, which neither eradicated tumors nor prevented recurrence. Similarly, DMGTF and MGTF without MW irradiation exhibited insufficient degradation and slow therapeutic onset, failing to restrain rapid tumor progression. In contrast, DMGTF under MW irradiation effectively eliminated tumor cells and suppressed tumor recurrence, enabling the most durable therapeutic benefit. Tumor-volume curves for individual mice (Fig. 5H) further illustrate the growth trajectories, confirming that DMGTF + MW, compared with other groups, not only minimized tumor burden but also extended survival. Body weights remained stable throughout the study, and no obvious pathological abnormalities were observed in the H&E staining of major organs (Fig. 5G and S11), suggesting good biocompatibility and tolerability.

### Pulmonary metastasis inhibition by microwave-activated DMGTF

**Fig. 6:**
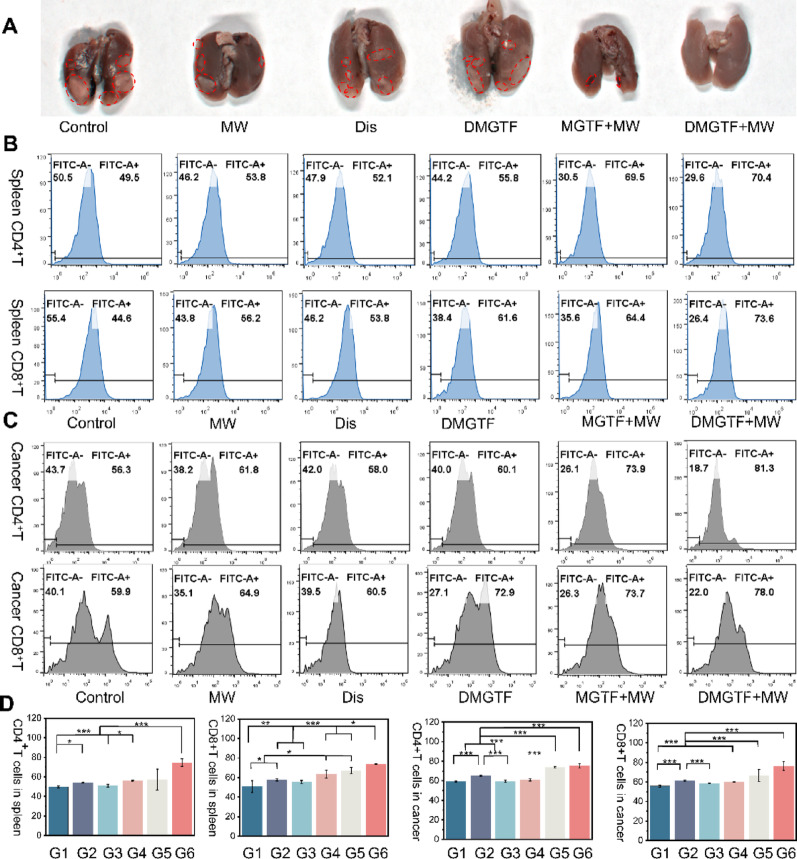
In vivo evaluation of DMGTF for metastatic tumor therapy. (**A**) Representative images of lungs collected from each treatment group. (**B-C**) Flow cytometric analysis of CD4⁺ and CD8⁺ T cells in spleens and cancer from each group (*n* = 3). (**D**) Quantitative comparison of CD4⁺/CD8⁺ T-cell populations in spleen and tumor tissues across different treatments. Data are presented as mean ± SD; *p < 0.05, **p < 0.01, ***p < 0.001

An H22 pulmonary metastasis model was established to evaluate the in vivo antitumor and survival efficacy of DMGTF (Fig. 6A). Upon microwave irradiation, tumor temperatures increased by 25.1 °C, 30.5 °C, and 33.2 °C in the MW, MGTF + MW, and DMGTF + MW groups, respectively (Figs. 6B-D), consistent with the subcutaneous tumor results. As shown in Fig. 6E and F, mice were euthanized at different time points in accordance with ethical guidelines when tumor burden reached defined endpoints. Both MGTF + MW and DMGTF + MW treatments effectively suppressed tumor growth and significantly prolonged survival, with all mice in the DMGTF + MW group surviving the full 14 days and exhibiting the smallest tumor volumes. Mice in the Control and Dis groups reached humane endpoints and were euthanized on day 9, whereas the DMGTF and MW-only groups were terminated on day 10 according to ethical requirements. These outcomes reflect the limited antitumor efficacy of Dis or MW alone, which neither eradicated tumors nor prevented recurrence. Similarly, DMGTF and MGTF without MW irradiation showed incomplete degradation and slow therapeutic onset, failing to counteract rapid tumor progression. Individual tumor growth trajectories (Fig. 6H) further confirmed that DMGTF + MW not only minimized tumor burden but also extended survival. Notably, while the MW-only group exhibited an initial reduction in tumor volume, rapid regrowth occurred after recurrence, highlighting the potential for tumor relapse following microwave therapy and emphasizing the need for combinatorial strategies to achieve durable treatment outcomes. Take together, these findings demonstrate that DMGTF, upon microwave activation, exhibits potent in vivo antitumor efficacy and markedly prolongs survival in H22-bearing mice. This effect can be attributed to the initial tumoricidal action of microwave irradiation, the dual blockade of tumor lactate metabolism mediated by diclofenac sodium and ROS, and the further cytotoxic contribution from immune cell activation induced by Mg²⁺.

### Antimetastatic and immunomodulatory effects of DMGTF

As shown in Figs. 6A and Figures S12, the control group exhibited the largest number of pulmonary metastatic nodules, which were densely distributed and partially fused into plaques. Both the Dis and DMGTF groups showed reduced metastases to varying degrees; however, slightly larger nodules were observed in the DMGTF group, likely due to the absence of microwave activation that limited nanoplatform performance. In contrast, the MW, MGTF + MW, and particularly the DMGTF + MW groups displayed markedly fewer nodules, with almost no visible lesions in the DMGTF + MW group, confirming that DMGTF effectively mediates microwave-enhanced inhibition of pulmonary metastasis. To further assess immune modulation, tumor and spleen tissues were analyzed by flow cytometry for CD4⁺ and CD8⁺ T-cell populations. As shown in Fig. 6B, C and D, the DMGTF + MW group exhibited the highest proportions of CD4⁺ and CD8⁺ T cells, increasing by up to 1.42- and 1.65-fold relative to the control, respectively. Here, G1-G6 correspond to the Control, MW, Dis, DMGTF, MGTF + MW, and DMGTF + MW groups, respectively. Furthermore, the functional status of intratumoral CD8⁺ T cells was evaluated by assessing IFN-γ expression. As shown in Figure S13, the DMGTF + MW group exhibited markedly increased IFN-γ expression, reaching 7.44-fold that of the control group, indicating enhanced cytotoxic T-cell activity. Taken together, these findings indicate that microwave-activated DMGTF not only suppresses primary tumor growth but also leverages its immune-activating capacity to robustly promote CD8⁺ and CD4⁺ T-cell expansion, thereby transforming immunosuppressive pressure into effective antitumor immune drive and ultimately inhibiting distant pulmonary metastases.

To further illustrate the overall therapeutic outcome, representative photographs documenting tumor growth, treatment response, and recurrence during the experimental process are summarized in Figure S14. As shown, tumors in the groups without microwave treatment grew rapidly and quickly reached the ethical endpoint. Although the MW-only and MGTF + MW groups exhibited partial tumor suppression at the early stage, recurrent tumors of varying sizes were frequently observed at the margins of the microwave-treated region during later stages. In contrast, the DMGTF + MW group showed only minimal tumor recurrence, with some mice even displaying complete suppression of tumor regrowth. These observations further demonstrate the superior ability of DMGTF under microwave activation to inhibit post-treatment tumor recurrence.

## Discuss

The proposed “dual-transformation” strategy involves metabolic modulation accompanied by immune activation. In this process, lactate changes from functioning primarily as an extracellular metabolic substrate to accumulating intracellularly and contributing to metabolic stress. Diclofenac sodium treatment together with ROS accumulation functionally suppresses MCT4-mediated lactate export, resulting in decreased extracellular lactate levels and increased intracellular accumulation. This observation supports a functional regulation of lactate transport rather than direct molecular inhibition of MCT4, and the precise regulatory mechanisms warrant further investigation. Meanwhile, the relatively elevated extracellular pH values (~7.7) observed in vitro represent relative alkalization under controlled culture conditions and do not necessarily replicate the absolute tumor microenvironment pH in vivo. In addition, previous studies have reported that magnesium ions participate in the regulation of immune cell proliferation and effector function. In this study, the DMGTF system promoted T-cell proliferation and IFN-γ secretion; however, the specific contribution of Mg²⁺ alone was not independently evaluated. Therefore, whether Mg²⁺ directly modulates mitochondrial respiration, membrane potential, or ATP production in T cells remains to be clarified. Further mechanistic studies will be required to delineate the role of magnesium in T-cell metabolic regulation. Collectively, these findings suggest coordinated metabolic and immune modulation and provide a basis for further mechanistic exploration.

## Conclusions

In summary, we propose a dual-transformation strategy to address the challenging problem of tumor recurrence and metastasis in microwave therapy. In this study, a microwave-and pH-dual-responsive, degradable MOF nanoplatform with liver-cancer-targeting capability has been developed to deliver diclofenac (Dis) and generate ROS for disrupting tumor lactic-acid metabolism, transforming lactic acid from a metabolic fuel into metabolic burden. While simultaneously relieving the immunosuppressive TME, DMGTF promotes T-cell proliferation and the secretion of IFN-λ and IFN-γ, thereby reactivating antitumor immunity, inducing long-term immune memory, and converting immune suppression into immune activation. Comprehensive in vitro and in vivo studies confirmed that the DMGTF nanoplatform not only strategically overcomes the off-target limitations of traditional lactate-metabolism inhibitors, but also effectively suppresses tumor growth and metastasis through this dual strategy. Overall, this work provides a promising approach to resolving tumor recurrence and metastasis after microwave therapy and provides a promising strategy that integrates lactic-acid blockade with immune reactivation for future clinical applications.

## Experimental section

### Materials

Gallium nitrate (Ga (NO₃)₃, 99.99%) and polyvinylpyrrolidone (PVP) were purchased from Shanghai Macklin Biochemical Co., Ltd. Magnesium nitrate, terephthalic acid, and ammonia solution were obtained from Sa’en Chemical Technology Co., Ltd. N, N-dimethylformamide (DMF, 99.5%) and ethanol (99.7%) were supplied by Beijing Chemical Reagents Company. All reagents were used as received without further purification. Deionized water was used in all aqueous preparations.

### Animals

All animal experiments were approved by the Experimental Animal Management Committee of the Technical Institute of Physics and Chemistry, Chinese Academy of Sciences (IACUC-IPC-23013) and conducted in accordance with institutional guidelines for the care and use of laboratory animals. Female BALB/c mice (18 ± 2 g) were used for all in vivo studies. For establishing the subcutaneous tumor model, H22 hepatocellular carcinoma ascites cells (2 × 10⁷) were suspended in 100 µL DMEM and injected into the right axilla of each mouse. Tumor growth was monitored until volumes reached approximately 100 mm³, at which point in vivo therapeutic or metastasis experiments were initiated.

### Synthesis of MgGa-MOF nanoparticles

MgGa-MOF nanoparticles were prepared via a solvothermal method. Gallium nitrate (22 mg), magnesium nitrate (7.1 mg), and PVP (100 mg) were dissolved in 10 mL DMF under sonication until a clear solution was obtained. Separately, 54 mg of terephthalic acid was dispersed in 10 mL DMF with ultrasonication. A mixture of 80 mL DMF and 30 µL ammonia solution was prepared, forming a milky suspension. The terephthalic acid solution was slowly added dropwise into the ammonia-containing DMF, during which the suspension gradually became lighter in color. Subsequently, the metal/PVP solution was added dropwise into the mixture, further lightening the suspension. An additional 100 mL of DMF was introduced, and the final solution was stirred thoroughly. The reaction mixture was transferred into a 50 mL Teflon-lined autoclave and heated at 150 °C for 10 h. After cooling to room temperature, the precipitates were collected by centrifugation (10,000 rpm, 5 min), washed three times with ethanol to remove residual solvents and unreacted reagents, and dried to yield MgGa-MOF nanoparticles.

### Synthesis of Dis @MgGa-MOF and DMGTF nanoparticles

For drug loading, MgGa-MOF (10 mg) was dispersed in 2 mL anhydrous ethanol, and diclofenac sodium (20 mg) was added. The mixture was stirred at room temperature for 6 h to allow efficient adsorption of the drug. The resulting Dis@MgGa-MOF nanoparticles were collected by centrifugation, washed three times with ethanol, and dried. To prepare DMGTF, Dis@MgGa-MOF nanoparticles (10 mg) were suspended in 2 mL anhydrous ethanol containing 1-tetradecanol (10 mg L⁻¹) under ultrasonication. Folic acid (1 mg) was dissolved in 3 mL methanol with ultrasonication, and 1 mL of this folic acid solution was added dropwise to the nanoparticle suspension. The mixture was ultrasonicated to ensure uniform coating and interaction. The solvent was removed under vacuum using a rotary evaporator, and the resulting yellowish-white powder was washed with deionized water, centrifuged at 10,000 rpm for 5 min, and collected as DMGTF nanoparticles.

### In vitro degradation

MGTF nanoparticles were dispersed in either saline or PBS (pH 5.7) to simulate physiological and tumor-like microenvironments, respectively. Suspensions were incubated at 37 °C with gentle shaking. At predetermined time points (3, 6, and 9 h), nanoparticles were collected by centrifugation for characterization. For microwave-treated samples, additional collections were performed after 10, 20, and 30 min of irradiation. Morphological changes and structural integrity were examined using transmission electron microscopy (TEM) to assess degradation behavior under different conditions.

### Microwave-responsive thermal and catalytic properties

Nanoparticle suspensions (0–15 mg mL⁻¹) in 1 mL saline were transferred to quartz dishes and irradiated at 1.8 W for 5 min. Temperature changes were monitored using an infrared thermal imaging camera at 10 s intervals. To evaluate intracellular ROS generation, four groups were tested: PBS, MW, PBS + MGTF, and PBS + MGTF + MW. Materials were incubated with 1 mg mL⁻¹ MGTF for 1 h, and ROS levels were determined using DCFH-DA, with fluorescence intensity measured at 520 nm.

### Drug release study

Dis release from DMGTF nanoparticles (5 mg mL⁻¹) was investigated under three conditions: PBS pH 7.4, PBS pH 5.7, and PBS pH 5.7 with microwave irradiation (1.8 W, 5 min). Samples were incubated at 37 °C in a shaking water bath. At 1, 3, 6, and 12 h, supernatants were collected after centrifugation and analyzed using UV-vis spectrophotometry at 276 nm. Cumulative drug release was calculated based on a standard calibration curve.

### In vitro cytotoxicity

L929, HepG2, and H22 cells were seeded in 96-well plates and treated with MGTF nanoparticles at concentrations of 0-200 µgmL⁻¹ for 24 h. Subsequently, 20µL of MTT solution was added, and cells were incubated for 4 h. The supernatant was removed, and 150µL DMSO was added to dissolve formazan crystals. Absorbance was measured using a microplate reader, and cell viability was calculated relative to untreated controls.

### In vitro therapeutic evaluation

HepG2 and H22 cells were treated with seven groups (Control, MW, Dis, MGTF, MGTF + MW, DMGTF, DMGTF + MW) at 100 µg mL⁻¹ for 24 h. Microwave groups were irradiated for 5 min and then reseeded into 96-well plates, while untreated groups were directly reseeded. Cell viability was assessed after 24 h using MTT and CCK-8 assays, measuring absorbance to evaluate the therapeutic effects of DMGTF with or without MW treatment.

### Cellular internalization and targeting

For internalization studies, MGTF nanoparticles were labeled with rhodamine and incubated with HepG2 cells (100 µg mL⁻¹) for 3, 6, 9, and 12 h. Uptake was visualized using confocal laser scanning microscopy (CLSM). The targeting ability of DMGTF was evaluated by hyperspectral imaging. DMGT or DMGTF (1 mg/mL, 1µL) was deposited on glass coverslips and sealed to build spectral libraries using a Cytoviva push-broom system. HepG2 cells were seeded onto the coverslips and incubated with 100 µg mL⁻¹ nanoparticles for 6 h, washed, fixed with 4% neutral formaldehyde, and imaged to assess folate-mediated cellular uptake.

### MCT4 inhibition and lactate/extracellular pH assays

HepG2 cells were treated with 100 µg mL⁻¹ of control, MW, Dis, DMGTF or MGTF + MW, DMGTF + MW for 6 h. Cells were fixed in pre-chilled ethanol, permeabilized with 0.2% Triton X-100, blocked with 1% BSA for 1 h, and incubated with CL488-22787 primary antibody (1:200) for 1.5 h at room temperature. Fluorescence imaging was used to evaluate MCT4 expression. For lactate secretion, cells were treated similarly, and extracellular lactate was measured using a commercial assay kit. Extracellular pH of the medium was also recorded to assess the effect of DMGTF on TME acidity.

### Intracellular ROS and mitochondrial damage

Cells were treated with formulations (100 µg mL⁻¹) for 6 h, and H₂O₂ (100µM) was used as a positive control. MW groups were irradiated for 5 min. ROS generation was measured using DCFH-DA, and mitochondrial membrane potential was assessed with 10µM JC-1 probe. Cells were washed and imaged under fluorescence microscopy to visualize mitochondrial integrity and oxidative stress.

### Co-culture with immune cells

HepG2 cells were cultured alone or with CTLL-2 immune cells and treated with formulations as previously described. Scratch assays were performed at the time of treatment, and wound closure was observed after 48 h using fluorescence microscopy. CTLL-2 cells were analyzed by flow cytometry to quantify cell number and expression of IFN-γ and CD69, evaluating immune activation induced by DMGTF treatments.

### In vivo therapeutic evaluation

Tumor-bearing mice (~100 mm³) were randomly assigned to six groups (control, MW, Dis, DMGTF, MGTF + MW, DMGTF + MW). Nanoparticles (100 mg·kg⁻¹) were administered intravenously. MW-treated groups received irradiation 6 h post-injection. Tumor volumes and body weights were monitored every day. At study endpoint, tumors and major organs were collected for histological analysis to evaluate treatment efficacy and biocompatibility.

### In vivo anti-metastasis study

Metastatic models were established by intravenous injection of 1 × 10⁵ 4T1 cells into H22 tumor-bearing mice. Mice were randomly assigned to six groups (*n* = 4). Nanoparticles were administered via tail vein, and MW-treated groups received 5 min irradiation (1.8 W) of primary tumors 6 h post-injection. Body weight and tumor growth were monitored over 14 days. At the endpoint, mice were sacrificed, and tumors and lungs were collected for weighing, imaging, metastatic nodule counting, histology, and immunological analysis.

## Supplementary Information

Below is the link to the electronic supplementary material.

Supplementary Material 1.

## Data Availability

All data are available in the main text, supportinginformation, and are also on request from the corresponding author.
