# Supplementary material for: A dual-transformable MgGa-MOF nanoplatform for HCC therapy via lactate metabolism blockade and immune reactivation
Source: J Nanobiotechnology. 2026 Apr 5;24:452. doi: 10.1186/s12951-026-04356-8 (PMC13200337; doi:10.1186/s12951-026-04356-8)
Supplement: Supplementary file 1 — Supplementary Material 1. [file 12951_2026_4356_MOESM1_ESM.docx]

**Supplementary Information**

A Dual-Transformable MgGa-MOF Nanoplatform for HCC Therapy via Lactate Metabolism Blockade and Immune Reactivation

Supplementary Figures

Figure S1. Stability Evaluation of Mg-Ga-MOF under Various Conditions.

a

b

Figure S2. (a) UV Absorption Curves of Diclofenac Sodium at Different Concentrations, (b) UV Absorption Standard Curve of Diclofenac Sodium.

b

a

Figure S3 (a) Microwave-induced temperature elevation curves of DMGTF different concentrations. (b) Comparison of temperature increments between DMGTF and MG under microwave irradiation.

Figure S4. Dose-dependent cytotoxicity of DMGTF toward HepG2 cells after 24 h incubation.

a

a

Figure S5. (a, b) In vivo acute toxicity evaluation in mice, including body weight monitoring and hematological analysis. (n=4)

Figure S6. In vivo acute toxicity evaluation in mice by H&E staining of major organs. (scales bar: 50μm)

Figure S7. Cellular Therapeutic Evaluation of DMGTF against H22 Cells. The mean ± standard deviation (SD) data are presented, with n>3, *p<0.05, **p<0.01 and ***p<0.001.

Figure S8. Intracellular lactate concentrations under different treatment conditions.

Figure S9. Co-incubation of HepG2 and CTLL-2 Cells: Stimulatory Effect of DMGTF on CD69 Secretion by CTLL-2. (n=3)

Figure S10. Flow cytometry analysis of CD69 expression in CTLL-2 cells after treatment with DMGTF alone.

Figure S11. H&E staining of major organs (heart, liver, spleen, lung, kidney, and tumor) in mice after different treatments. (scales bar: 50)

Figure S12. Quantification of the average number of lung nodules in different treatment groups (n = 3).

Figure S13. Flow cytometry analysis of IFN-γ expression in intratumoral CD8⁺ T cells under different treatment conditions.

Figure S14. Representative photographs of tumor growth and recurrence in different treatment groups.
